# Machine-learning algorithms for forecast-informed reservoir operation (FIRO) to reduce flood damages

**DOI:** 10.1038/s41598-021-03699-6

**Published:** 2021-12-21

**Authors:** Manizhe Zarei, Omid Bozorg-Haddad, Sahar Baghban, Mohammad Delpasand, Erfan Goharian, Hugo A. Loáiciga

**Affiliations:** 1 Department of Irrigation & Reclamation Engineering, Faculty of Agriculture Engineering & Technology, College of Agriculture & Natural Resources, University of Tehran, 3158777871 Karaj, Iran; 2 Civil and Environmental Engineering, College of Engineering and Computing, University of South Carolina, 300 Main St. Room C206, Columbia, SC 29208 (803) 777 4625 USA; 3 Department of Geography, University of California, Santa Barbara, CA 93016-4060 USA

**Keywords:** Climate sciences, Ecology, Environmental sciences, Environmental social sciences, Hydrology, Natural hazards, Engineering, Mathematics and computing

## Abstract

Water is stored in reservoirs for various purposes, including regular distribution, flood control, hydropower generation, and meeting the environmental demands of downstream habitats and ecosystems. However, these objectives are often in conflict with each other and make the operation of reservoirs a complex task, particularly during flood periods. An accurate forecast of reservoir inflows is required to evaluate water releases from a reservoir seeking to provide safe space for capturing high flows without having to resort to hazardous and damaging releases. This study aims to improve the informed decisions for reservoirs management and water prerelease before a flood occurs by means of a method for forecasting reservoirs inflow. The forecasting method applies 1- and 2-month time-lag patterns with several Machine Learning (ML) algorithms, namely Support Vector Machine (SVM), Artificial Neural Network (ANN), Regression Tree (RT), and Genetic Programming (GP). The proposed method is applied to evaluate the performance of the algorithms in forecasting inflows into the Dez, Karkheh, and Gotvand reservoirs located in Iran during the flood of 2019. Results show that RT, with an average error of 0.43% in forecasting the largest reservoirs inflows in 2019, is superior to the other algorithms, with the Dez and Karkheh reservoir inflows forecasts obtained with the 2-month time-lag pattern, and the Gotvand reservoir inflow forecasts obtained with the 1-month time-lag pattern featuring the best forecasting accuracy. The proposed method exhibits accurate inflow forecasting using SVM and RT. The development of accurate flood-forecasting capability is valuable to reservoir operators and decision-makers who must deal with streamflow forecasts in their quest to reduce flood damages.

## Introduction

Floods are natural hazards that affect an average of 80 million people annually and cause more deaths and financial losses than any other natural disaster^1,2^. One of the traditional ways to control floods is building dams and reservoirs, which are operated to create flood control space to store and regulate high flows. Water is released gradually according to the safe discharge in the rivers downstream to meet the required flood control space. Accurate forecasts of reservoir inflows must be made before the flood events. Identifying appropriate algorithms for forecasting future reservoir inflow is paramount to reservoir operators. An example of Forecast-Informed Reservoir Operation (FIRO) has been practised in Mendocino Lake, California, during the past few decades^3^. FIRO is a strategy that improves informed decisions about releasing water from reservoirs and increases flexibility in the operation and management of reservoirs by improving hydrologic forecasting^3,4^.

Physically-based and statistical models have been applied to forecast reservoir inflows^5^. Physically-based models simulate the involved hydrological processes and estimate reservoir inflow^6–8^. Physically-based models such as the Soil and Water Assessment Tool (SWAT)^9^, the watershed-scale Long-Term Hydrologic Impact Assessment model (watershed-scale L-THIA)^10^ and the Hydrological Simulation Program—Fortran (HSPF)^11^ are used to simulate water cycle components^12^. Physically-based models can be applied to simulate flood events accounting for the key hydrologic processes involved. They often require large volumes of hydro-geomorphological data, detailed information about the characteristics and dynamic changes of a watershed, and are computationally expensive^13^. Besides, physically-based models make simplifications of hydrologic processes^14^ and involve parameters that must be calibrated, sometimes with in-depth effort, which causes model forecasts to vary greatly among models^15^.

Recent advancements in Machine Learning (ML) modeling techniques can address and overcome the difficulties that beset physically-based models, giving impetus to using data-driven algorithms and ML modeling in reservoir inflow forecasting, among others. ML algorithms can be applied to forecast reservoir inflow by relying on relevant data rather than simulating the hydrological processes involved^16^. The advantages of using ML algorithms are easier and faster implementation, less computational effort, and reduced complexity compared to the physically-based models, particularly the distributed type variety^17,18^. A variety of ML algorithms have been applied to analyze big data and large-scale systems, in particular for hydrologic modeling and water resources management^19–23^. For example, Support Vector Machine (SVM) was implemented for lake water level forecast^24^, modelling daily reference evapotranspiration^25^, soil moisture estimation^26^, water quality forecast modelling^27^, and groundwater quality characterization^28^. Artificial Neural Networks (ANNs) were applied to forecasting the runoff coefficient^29^, river discharges forecasting^30^, water demand forecasting under climate change^31^, wastewater temperature forecasting^32^, and groundwater level simulation^33^. Genetic Programming (GP) was applied to forecasting rainfall-runoff response^34^, suspended sediment modeling^35^, calculating of the optimal operation of an aquifer-reservoir system^36^, modelling of groundwater^37^, and crop yield estimating^38^.

Several previous studies have forecasted river flow for flood routing^39^, flood susceptibility mapping^40^ and calculating flood damages^41^ in unregulated rivers. This work proposes a river flow forecasting method to improve flood mitigation by reservoirs and guide FIRO to reduce flood damages.

Heavy and continuous precipitation in 2019 led to severe floods in large areas of Iran, which caused great material and human losses. The southwestern basins of the country had the most share of precipitation and suffered significant damages due to floods. River flow forecasts did not forecast accurately the magnitude of the reservoirs inflow, which led to inadequate flood control by reservoirs operation^42^. The 2019 flood event raised questions about the poor river flow forecasting performance. This work addresses these questions. This work develops methods for flood forecasting in terms of timing and magnitude to allow operators to release water from reservoirs and route the floods with minimal or no damage. The flood forecasts are made with 1- and 2-month time-lag patterns in the algorithms. Each time-lag pattern produces four flood projections, which correspond to the wettest months in the study area. Specifically, the flood forecasts provide operators with information about the reservoirs inflows likely to occur during the wettest months of the year (January, February, March and April) with one month lead time (obtained with the forecasts based on the 1-month time-lag pattern) and with two months lead time (obtained with the forecasts based on the 2-month time-lag pattern). This study’s flood forecasting methodology considers the effect that practical limitations, such as data scarcity, have on the accuracy of the forecasts. A challenge in developing countries is the scarcity of hydro-climate data due to the lack of modern hydrologic and weather monitoring stations. This paper’s data-driven flood forecasting methodology is intended to support FIRO and reduce flood damages.

## Methods

**Figure 1:**
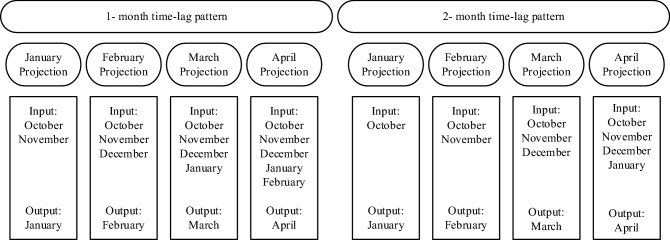
Schematic of projections of 1-month and 2-month time-lag patterns.

**Figure 2:**
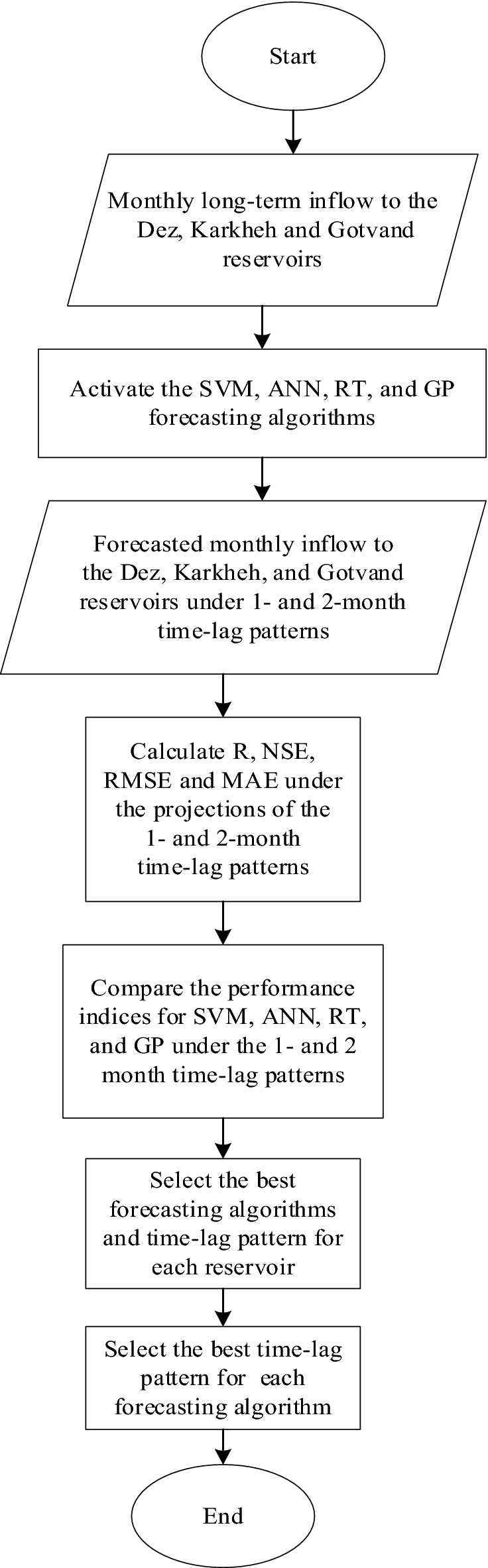
Flowchart of this study’s methodology.

This study applies the SVM, ANN, RT, and GP, for forecasting monthly reservoirs inflow with 1- and 2-month time lags. The historical data for inflow to the Dez, Karkheh, and Gotvand reservoirs were collected and used to build the ML algorithms. The inputs to the algorithms for the Dez, Karkheh, and Gotvand reservoirs are the monthly inflows for 1965–2019, 1957–2019, and 1961–2019, respectively. Four projections were designed for the 1-month time lag and the 2-month time lag patterns based on the input and output months, as depicted in Fig. 1. Figure 2 displays the flowchart of this paper’s methodology.

### Support vector machine

**Figure 3:**
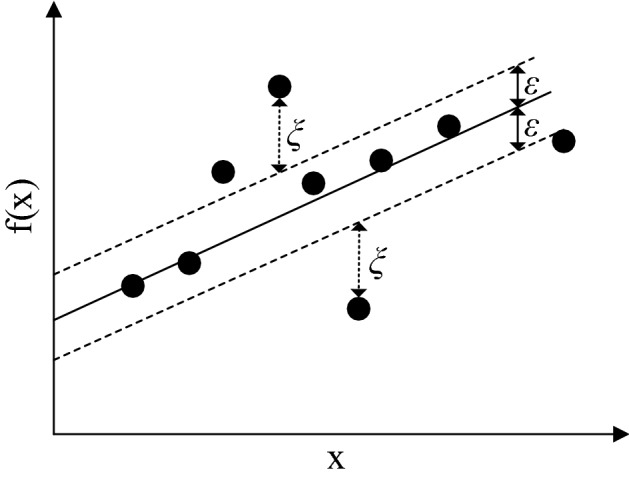
Illustration of the error function of SVR.

Support Vector Machine was introduced by Vapnik et al.^43^. SVM performs classification and regression based on statistical learning theory^44^. The regression form of SVM is named support vector regression (SVR). Vapnik et al.^45^ defined two functions for SVR design. The first function is the error function. (Eq. (1), see Fig. 3). The second function is a linear function that calculates output values for input, weight, and deviation values (Eq. 2):

1 $$ \left| {y - f\left( x \right)} \right| = \left\{ {\begin{array}{ll} 0 & {if\;\left| {y - f\left( x \right)} \right| \le \varepsilon } \\ {\left| {y - f\left( x \right)} \right| - \varepsilon = \xi } & {otherwise} \\ \end{array} } \right. $$

2 $$ f\left( x \right) = W^{T} x + b $$

where $y$, $f(x)$, $\varepsilon$, $\xi$, $W$, $b$, $T$ denote respectively the observational value, the output value calculated by SVR, a function sensitivity value, a model penalty, the weight applied to the variable $x$, the deviation of $W^{T} x$ from the $y$, and the vector/matrix transpose operator.

It is seen in Fig. 3 that the first function (Eq. 1) does not apply a penalty to the points where the difference between the observed value and the calculated value falls within the range of $( - \varepsilon , + \varepsilon )$. Otherwise, a penalty $\xi$ is applied. SVR solves an optimization problem that minimizes the forecast error (Eq. 3) to improve the model’s forecast accuracy. Equations (4) and (5) represent the constraints of the optimization problem.

3 $$  minimize\frac{1}{2}\left\| W \right\|^{2}  + C\sum\limits_{{i = 1}}^{m} {\left( {\xi _{i}^{ - }  + \xi _{i}^{ + } } \right)}   $$

Subject to:

4 $$ (W^{T} x + b) - y_{i} < \varepsilon + \xi_{i}^{ + } \;\;\;i = 1, \, 2, \ldots , \, m $$

5 $$ y_{i} - \left( {W^{T} x + b} \right) \le \varepsilon + \xi_{i}^{ - } \;\;\;i = 1, \, 2, \ldots , \, m $$

where $C$, *m*, $\xi_{i}^{ - }$, $\xi_{i}^{ + }$, $y_{i}$, and || || denote respectively the penalty coefficient, the number of input data to the model in the training phase, the penalty for the lower bound $( - \varepsilon , + \varepsilon )$, the penalty for the upper bound $( - \varepsilon , + \varepsilon )$, the *i-th* observational value, and vectorial magnitude. The values of *W* and *b* are calculated by solving the optimization problem embodied by Eqs. (3)–(5) with the Lagrange method, and they are substituted in Eq. (2) to calculate the SVR output. SVR is capable of modeling nonlinear data, in which case it relies on transfer functions to transform the data to such that linear functions can be fitted to the data. Reservoirs inflow is forecasted with SVR was performed with the Tanagra software. The transfer function selected and used in this study is the Radial Basis Function (RBF), which provided better results than other transfer functions. The weight vector W is calculated using the Soft Margin method^46^, and the optimal values of the parameters $\xi_{i}^{ - } , + \xi_{i}^{ + }$ and C were herein estimated by trial and error.

### Regression tree (RT)

RT involves a clustering tree with post-pruning processing (CTP). The clustering tree algorithm has been reported in various articles as the forecasting clustering tree^47^ and the monothetic clustering tree^48^. The clustering tree algorithm is based on the top-down induction algorithm of decision trees^49^; This algorithm takes a set of training data as input and forms a new internal node, provided the best acceptable test can be placed in a node. The algorithm selects the best test scores based on their lower variance. The smaller the variance, the greater the homogeneity of the cluster and the greater the forecast accuracy. If none of the tests significantly reduces the variance the algorithm generates a leaf and tags it as being representative of data^47,48^.

The CTP algorithm is similar to the clustering tree algorithm, except that its post-pruning process is performed with a pruning set to create the right size of the tree^50^.

RT used in this study is programmed in the MATLAB software. The minimum leaf size, the minimum node size for branching, the maximum tree depth, and the maximum number of classification ranges are set by trial and error in this paper’s application.

### Genetic programming (GP)

**Figure 4:**
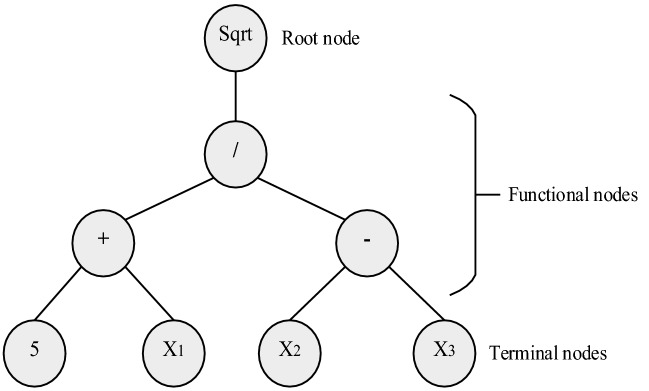
Example of mathematical relations produced by GP based on a tree representation for the function:$f\left( {X_{1} , X_{2} ,X_{3} } \right) = \left( {5 X_{1} /\left( {X_{2} X_{3} } \right)} \right)^{2}$.

GP, developed by Cramer^51^ and Koza^52^, is a type of evolutionary algorithm that has been used effectively in water management to carry out single- and multi-objective optimization^53^. GP finds functional relations between input and output data by combining operators and mathematical functions relying on structured tree searches^44^. GP starts the searching process by generating a random set of trees in the first iteration. The tree's length creates a function called the depth of the tree which the greater the depth of the tree, the more accurate the GP functional relation is^54^. In a tree structure, all the variables and operators are assumed to be the terminal and function sets, respectively. Figure 4 shows mathematical relational functions generated by GP. Genetic programming consists of the following steps:

- Select the terminal sets: these are the problem-independent variables and the system state variables.
- Select a set of functions: these include arithmetic operators (÷ , ×, −, +), Boolean functions (such as "or" "and"), mathematical functions (such as sin and cos), and argumentative expressions (such as if–then-else), and other required statements based on problem objectives.
- Algorithmic accuracy measurement index: it determines to what extent the algorithm is performing correctly.
- Control components: these are numerical components, and qualitative variables are used to control the algorithm's execution.
- Stopping criterion: which determines when the execution of the algorithm is terminated.

The Genexprotools software was implemented in this study to program GP. The GP parameters, operators, and linking functions were chosen based on the lowest RMSE in this study. The GP model's parameters and operators applied in this study are listed in Table 1.

**Table 1:** Operators and range of parameters used in GP.

	Range or type of parameters	The best case
Number of chromosomes	20–120	100
Number of genes	3–20	15
Generation	2000–5000	2000
Crossover	0–1	0.9
Mutation	0–1	0.1
Operators used	+/−/÷/x/sin/cos/sqrt/x^3^/x^2^/exp/tan/and/or/if	+/−/÷/x/sin/cos/sqrt/x^2^
Stopping criteria	Generation number / Random / Best fitness	Best fitness

#### Artificial Neural Network (ANN)

ANN, developed by McCollock and Walterpits^55^, is an artificial intelligence-based computational method that features an information processing system that employs interconnected data structures to emulate information processing by the human brain^56^. A neural network does not require precise mathematical algorithms and, like humans, can learn through input/output analysis relying on explicit instructions^57^. A simple neural network contains one input layer, one hidden layer, and one output layer. Deep-learning networks have multiple hidden layers^58^. ANN introduces new inputs to forecast the corresponding output with a specific algorithm after training the functional relations between inputs and outputs.

**Figure 5:**
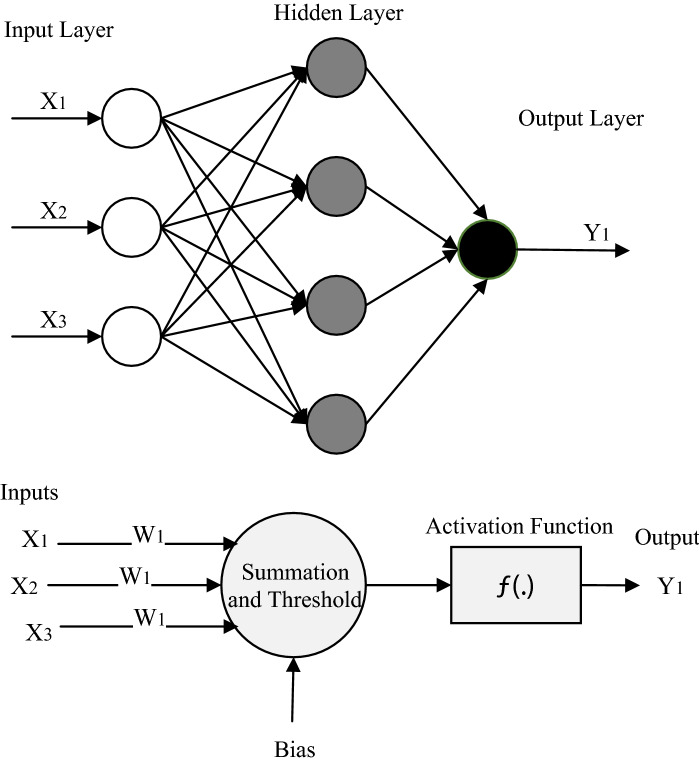
The general structure of a three-layer feed forward ANN and processing architecture.

This study applies the Multi-Layer Perceptron (MLP). A three-layer feed-forward ANN that features a processing element, an activation function, and a threshold function, as shown in Fig. 5. In MLP, the weighted sum of the inputs and bias term is passed to activation level through a transfer function to create the one output.

The output is calculated with a nonlinear function as follows:

6 $$ Y = f\left( {\mathop \sum \limits_{i = 1}^{n} W_{i} X_{i} + b} \right) $$

where $W_{i}$, $X_{i}$, $b$, $f$, and $Y$ denote the i-th weight factor, the i-th input vector, the bias, the conversion function, and the output, respectively.

The ANN was coded in MATLAB. The number of epochs, the optimal number of hidden layers, and the number of neurons of the hidden layers were found through a trial-and-error procedure. The model output sensitivity was assessed with various algorithms; however, the best forecasting skill was achieved with the Levenberg–Marquardt (LM) algorithm^59^, and the weight vector W is calculated using the Random Search method^60^. Furthermore, the Tangent Sigmoid and linear transfer function were chosen by trial and error and used in the hidden and output layers, respectively.

70% of the total data were randomly selected and used for training SVM, ANN, RT, and GP. The remaining 30% of the data were applied for testing the forecasting algorithms.

### Performance-evaluation indices

The forecasting skill of the ML algorithms (SVM, ANN, RT, and GP) was evaluated with the Correlation Coefficient (R), the Nash–Sutcliffe Efficiency (NSE), the Root Mean Square Error (RMSE), and the Mean Absolute Error (MAE) in the training and testing phases. The closer the R and NSE values are to 1, and the closer the RMSE and MAE values are to 0, the better the performance of the algorithms^20^. Equations (7)–(10) describe the performance indices:

7 $$ NSE = 1 - \frac{{\mathop \sum \nolimits_{i = 1}^{n} \left( {Q_{fore,\;i} - Q_{obs,i} } \right)^{2} }}{{\mathop \sum \nolimits_{i = 1}^{n} \left( {Q_{obs,i} - Q_{mean \; obs} } \right)^{2} }} $$

8 $$ R = \frac{{\mathop \sum \nolimits_{i = 1}^{n} \left( {Q_{fore,i} - Q_{mean \; fore} } \right)\left( {Q_{obs,i} - Q_{mean \; obs} } \right)}}{{\sqrt {\mathop \sum \nolimits_{i = 1}^{n} \left( {Q_{fore,i} - Q_{mean \; fore} } \right)^{2} } \sqrt {\mathop \sum \nolimits_{i = 1}^{n} \left( {Q_{obs,i} - Q_{mean \; obs} } \right)^{2} } }} $$

9 $$ MAE = \frac{1}{n}\mathop \sum \limits_{i = 1}^{n} \left| {Q_{fore,i} - Q_{obs,i} } \right| $$

10 $$ RMSE = \sqrt {\frac{{\mathop \sum \nolimits_{i = 1}^{n} \left( {Q_{fore,i} - Q_{obs,i} } \right)^{2} }}{n}} $$

in which $$ Q_{fore,i}$$, $$Q_{obs,i}$$, $$Q_{mean \; fore}$$, $$Q_{mean \; obs}$$, $$i$$, and $$n$$ denote the forecasted inflow, observed inflow, mean forecasted inflow, mean observed inflow, time step, and the total number of time steps during training and testing phases, respectively.

### Ethics approval

All authors complied with the ethical standards.

### Consent to participate

All authors consent to participate.

### Consent for publish

All authors consent to publish.

## Case study

The Great Karun Basin, Iran, is part of the Persian Gulf catchment. It is located in southwestern Iran, with an area of about 67,257 km^2^. The main river of the basin, the Karun, with a length of about 950 km, stems from the Yellow Mountains and flows through mountainous areas in Indika and Masjed Soleyman and ultimately discharges into the Persian Gulf. Dez and Gotvand are the two main reservoirs which are located in this basin.

**Figure 6:**
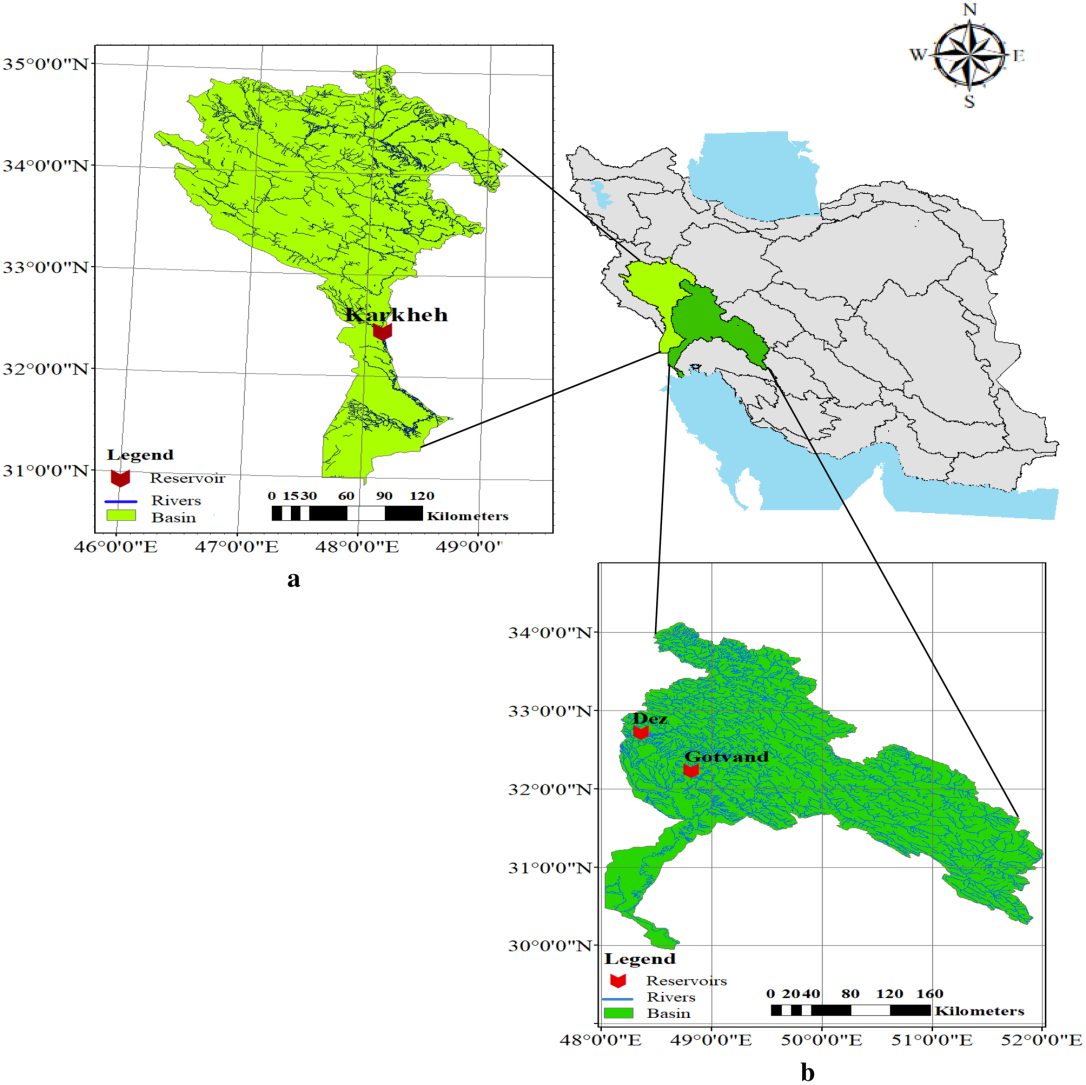
Location of the study areas in Iran; (**a**) Karkheh basin and (**b**) Great Karun basin (ArcGIS 10.8.1).

Karkheh Basin is located in western Iran, in the middle and southwestern regions of the Zagros Front. The area of this basin is about 51,604 km^2^. Karkheh reservoir is located in this basin. Table 2 lists the characteristics of the Dez, Karkheh, and Gotvand reservoirs. Figure 6 shows the location of Dez and Gotvand reservoirs in the Great Karun basin and the Karkheh reservoir in the Karkheh basin.

**Table 2:** Specifications of the Dez, Karkheh and Gotvand reservoirs.

Reservoirs	Reservoir Type	Capacity (× 10^6^ m^3^)	Height (m)	River	Purposes	Basin	Location
Dez	Double arched concrete	2698	203	Dez River	Hydropower, flood control, irrigation water	Great Karun	Khuzestan
Karkheh	Soil with clay core	5900	127	Karkheh River	Hydropower, Industrial, agricultural and drinking supply	Karkheh	Khuzestan
Gotvand	Gravel and clay core	4500	760	Karun River	Hydropower, flood control, agricultural water supply	Great Karun	Khuzestan

During March and April 2019 Iran faced three major waves of extreme precipitation, leading to extreme floods with long return periods in large parts of Iran^61,62^. Before the 2019 flood many parts of Iran suffered drought and the drying of lakes and rivers for almost 30 years due to climatic change^63^. The southwestern regions of Iran including Great Karun and Karkheh basins endured the brunt of the second and third waves of precipitation and suffered severe damages due to fluvial floods.The Dez, Gotvand and Karkheh reservoirs received large volumes and precipitation and river flows. Table 3 shows the average, minimum, and maximum inflows to the Dez, Karkheh, and Gotvand reservoirs during January through April. This study develops a method to forecast reservoirs inflows in the Great Karun and Karkheh basins, which can be applied to future events.

**Table 3:** Minimum, maximum and average inflow to Dez, Karkheh and Gotvand reservoirs in January until April 2019.

Reservoir	Min inflow (m^3^/s)	Max inflow (m^3^/s)	Average inflow (m^3^/s)
Dez	1080.205	4509.307	2217.165
Karkheh	1067.600	5539.832	2412.184
Gotvand	1326.415	4640.422	2496.173

## Results and discussion

### Dez reservoir evaluation

The values of the performance indices for SVM, ANN, RT and GP with the time-lag patterns in the Dez reservoir are listed in Table 4. It is seen that SVM had minimal RMSE, and RT had minimal MAE with the 1-month time-lag pattern applied to the January and April projections. SVM and RT performed better than the other algorithms in the testing phase. SVM had the best RMSE (MAE), 74.27 (74.26) for the February projection. RT achieved the best results for the March projection by having RMSE (MAE) of 33.37 (8.34). Appendix 1 presents the performance of the applied forecasting algorithms corresponding to the 1-month time-lag pattern for the Dez reservoir for the four projections.

**Table 4:** Results of the applied algorithms obtained with the 1-month and 2-month time-lag patterns in Dez reservoir.

Algorithms	Phase	Projections	1-month time-lag pattern				2-month time-lag pattern			
			R	NSE	RMSE (m^3^/s)	MAE (m^3^/s)	R	NSE	RMSE (m^3^/s)	MAE (m^3^/s)
SVM	Train	January	0.98	0.96	66.91	59.49	0.56	0.28	291.27	190.36
		February	0.98	0.97	73.45	71.82	0.95	0.89	137.48	136.8
		March	0.97	0.93	159.2	151.87	0.93	0.97	170.36	168.1
		April	0.97	0.91	216.2	206	0.91	0.97	217.4	209.7
	Test	January	0.98	0.97	66.76	61.51	0.55	0.17	375.528	263.67
		February	0.99	0.98	74.27	74.26	0.97	0.94	146.67	144.15
		March	0.97	0.89	171.13	170.02	0.96	0.9	166.37	163.81
		April	0.99	0.95	214.1	203.25	0.99	0.95	224.14	221.14
ANN	Train	January	0.87	0.87	169.4	155.4	0.45	0.8	316.96	198.2
		February	0.58	0.81	423.78	303.4	0.57	-0.03	396.52	421.6
		March	0.84	0.76	374.27	321.3	0.73	0.87	602.68	351.6
		April	0.92	0.97	315.63	229.7	0.77	0.91	464.32	536.1
	Test	January	0.81	0.78	140.49	236.8	0.62	0.63	370.52	342.4
		February	0.85	0.63	702.95	422.7	0.79	0.25	641.58	451.7
		March	0.51	0.76	531.84	203.3	0.9	0.31	304.56	419.7
		April	0.61	0	983.54	543	0.81	0.32	710.35	444.9
RT	Train	January	0.97	0.94	82.58	22.54	0.91	0.82	145.47	51.32
		February	0.94	0.96	146.33	38.75	0.92	0.84	173.55	139.36
		March	0.9	0.77	302.16	79.54	0.99	0.99	53.09	11.82
		April	0.94	0.87	269.07	61.28	0.98	0.98	108.96	26.82
	Test	January	0.94	0.89	138.55	51.98	0.85	0.81	281.07	83
		February	0.9	0.75	301.22	93.25	0.88	0.76	294.99	209.52
		March	0.99	0.99	33.37	8.34	0.93	0.84	209.24	81.65
		April	0.97	0.94	232.32	58.08	0.9	0.8	437.14	130.88
GP	Train	January	0.85	0.72	180.24	150.4	0.86	0.74	174.06	122.3
		February	0.89	0.78	200.93	156.1	0.89	0.95	258.72	203.3
		March	0.92	0.75	262.57	198.1	0.89	0.93	376.74	285.4
		April	0.88	0.77	348.69	270.2	0.89	0.95	339.29	274.7
	Test	January	0.83	0.52	493.03	353.2	0.87	0.72	259.43	222.1
		February	0.9	0.78	351.47	314.6	0.78	0.78	704.31	689.6
		March	0.87	0.86	360.63	327.3	0.87	0.77	597.18	489.1
		April	0.88	0.73	854.05	656.3	0.83	0.83	975.52	933.3

The results listed in Table 4 indicate that the RT’s RMSE (MAE) obtained with the 2-month time-lag pattern applied to the January projection is 181.07 (63), which means a better forecast than the other algorithms in the testing phase. SVM had the best RMSE (MAE), 146.67 (144.15) for the February projection. SVM had the best values of RMSE for the other projections, and RT had the lowest values of the MAE. The 2-month time-lag pattern results associated with the Dez reservoir are presented in Appendix 2.

### Karkheh reservoir evaluation

It is seen in Table 5 that SVM and RT have the best RMSE and MAE values, respectively, with the 1-month time-lag pattern applied to the January projection and the February projection and produced more accurate forecasts than the other algorithms. The smallest RMSE and MAE recorded in the testing phase corresponded to SVM for the other projections. The 1-month time-lag pattern results corresponding to the Karkheh reservoir under the four projections herein considered are presented in Appendix 3.

**Table 5:** Results of the applied algoriths obtained with the 1-month and 2-month time-lag patterns in Karkheh reservoir.

Algorithms	Phase	Projections	1-month time-lag pattern					2-month time-lag pattern		
			R	NSE	RMSE (m^3^/s)	MAE (m^3^/s)	R	NSE	RMSE (m^3^/s)	MAE (m^3^/s)
SVM	Train	January	0.98	0.97	36.88	36.7	0.66	0.39	172.9	108.71
		February	0.94	0.89	108.88	107.87	0.95	0.89	109.75	109.32
		March	0.98	0.94	117.48	109.55	0.97	0.94	118.88	112.91
		April	0.97	0.9	237.75	225.75	0.96	0.9	234.55	220.96
	Test	January	0.99	0.98	35.5	33	0.45	0.11	246.25	155.83
		February	0.97	0.92	110.44	110.3	0.97	0.93	128.51	119.52
		March	0.94	0.87	124.48	118.46	0.92	0.85	131.63	131.5
		April	0.99	0.96	247.57	235.46	0.98	0.96	241.08	233.11
ANN	Train	January	0.81	0.94	142.74	89.62	0.61	0.89	185.02	115.48
		February	0.84	0.84	213.89	205.7	0.73	0.83	267.32	192.73
		March	0.86	0.87	254.17	236.72	0.73	0.78	268.1	316.71
		April	0.89	0.89	331.79	351.23	0.92	0.87	384.32	385.78
	Test	January	0.9	0.67	120.21	101.82	0.83	0.1	127.03	207.09
		February	0.85	0.97	400.93	159.73	0.84	0.88	497.2	119.36
		March	0.85	0.75	530.11	115.76	0.82	0.66	241.06	120.79
		April	0.92	0.73	482.03	407.53	0.84	0.99	440.59	153.66
RT	Train	January	0.88	0.75	110.85	25.2	0.84	0.7	119.96	78.27
		February	0.92	0.84	131.46	31.86	0.9	0.82	139.97	53.55
		March	0.9	0.8	208.83	157.53	0.96	0.92	135.23	34
		April	0.88	0.78	359.25	245.1	0.94	0.88	261.51	69
	Test	January	0.97	0.96	52.82	16.98	0.87	0.84	105.09	83.34
		February	0.92	0.79	232.26	74.25	0.96	0.93	133.07	43.66
		March	0.89	0.79	157.8	137.77	0.9	0.79	156.5	47.58
		April	0.95	0.77	562.09	337.45	0.99	0.98	182	66
GP	Train	January	0.88	0.74	111.39	86.5	0.92	0.96	103.76	82.99
		February	0.89	0.8	146.99	109.91	0.89	0.96	144.02	103.5
		March	0.91	0.82	199.54	168.72	0.88	0.93	329.02	249.39
		April	0.91	0.82	318.21	257.65	0.9	0.81	336	246.47
	Test	January	0.92	0.83	114.86	74.92	0.87	0.63	485.13	380.42
		February	0.83	0.56	488.5	327.75	0.89	0.82	418.2	264.39
		March	0.92	0.62	308.95	231.78	0.88	0.86	239.58	217.05
		April	0.84	0.86	290.73	239.18	0.91	0.73	1312.2	667.64

The results in Table 5 indicate that RT had the best accuracy according to the RMSE and MAE values for the 2-month time-lag pattern in the testing phase for the January projection and April projection. The highest accuracy corresponded to SVM and RT according to the RMSE and MAE values, respectively, for the February and March projections. Appendix 4 presents the 2-month time-lag pattern results for the Karkheh reservoir with the applied forecasting algorithms.

### Gotvand reservoir evaluation

The SVM, RT, ANN, and GP results associated with the Gotvand reservoir are listed in Table 6. SVM and RT had the lowest RMSE and MAE values, respectively, for the January and April projections with the 1-month time-lag pattern in the testing phase. SVM produced the lowest RMSE (MAE), 93.46 (91.12) for the February projection. RT had the lowest RMSE (MAE), 257.91 (60.79) for the April projection. Appendix 5 presents the performance of the applied forecasting algorithms corresponding to the 1-month time-lag pattern for the four projections associated with the Gotvand reservoir.

**Table 6:** Results of the applied algorithms obtained with the 1-month and 2-month time-lag patterns in Gotvand reservoir.

Algorithms	Phase	Projections	1-month time-lag Pattern				2-month time-lag pattern			
			R	NSE	RMSE (m^3^/s)	MAE (m^3^/s)	R	NSE	RMSE (m^3^/s)	MAE (m^3^/s)
SVM	Train	January	0.95	0.91	168.55	161.33	0.58	0.3	463.1	264.83
		February	0.99	0.98	97.13	96.9	0.97	0.93	176.4	176
		March	0.96	0.94	259.37	243.3	0.94	0.94	268.06	259.17
		April	0.99	0.76	531.86	431.53	0.99	0.93	286.08	272.8
	Test	January	0.98	0.96	168.36	162.52	0.5	0.13	752.73	423.8
		February	0.99	0.97	93.46	91.12	0.94	0.92	152.67	29.4
		March	0.97	0.83	266.91	256.87	0.97	0.83	260	68.67
		April	0.99	0.86	451.71	328.71	0.97	0.94	260.76	118.59
ANN	Train	January	0.89	0.83	260.94	314.65	0.55	0.82	614.84	308.85
		February	0.67	0.78	475.36	314.65	0.5	0.8	628.36	324.96
		March	0.82	0.86	754.99	585.15	0.66	0.8	823.13	688.09
		April	0.98	0.93	213.67	585.15	0.8	0.93	689.69	549.31
	Test	January	0.75	0.78	608.82	356.91	0.77	0.82	262.02	324.96
		February	0.83	0.91	561.41	356.91	0.71	0.78	638.53	308.85
		March	0.81	0.78	1123.49	432.3	0.88	0.9	613.57	245.62
		April	0.83	0.85	1265.67	432.3	0.89	0.93	641.84	352.06
RT	Train	January	0.99	0.98	75.29	19.84	0.87	0.76	269.88	205.18
		February	0.98	0.97	125.33	32.79	0.94	0.88	231.38	73.84
		March	0.97	0.96	229.28	77	0.95	0.89	362.89	93.13
		April	0.93	0.86	400.06	118.1	0.9	0.83	462.5	134.4
	Test	January	0.87	0.75	405.71	145.92	0.94	0.89	256.84	209.1
		February	0.92	0.82	244.06	67.56	0.96	0.9	178.83	58.68
		March	0.93	0.84	257.91	60.79	0.91	0.8	290.64	93.05
		April	0.92	0.88	457.79	107.9	0.94	0.87	425.4	111.73
GP	Train	January	0.87	0.75	276.12	208.4	0.91	0.84	218.27	178.22
		February	0.91	0.83	269.21	222.73	0.9	0.81	292.16	250.08
		March	0.92	0.81	485.81	377.91	0.92	0.95	577.06	410.09
		April	0.89	0.75	528.3	418.58	0.92	0.98	500.43	407.2
	Test	January	0.87	0.89	687.57	555.22	0.83	0.79	1331	877.99
		February	0.86	0.89	662.93	515.28	0.87	0.93	415.37	332.16
		March	0.91	0.88	1396.59	1008.4	0.93	0.87	966.49	949.62
		April	0.91	0.69	788.68	624.91	0.83	0.75	3325	2587

It is seen in Table 6 that RT had the best RMSE (MAE) value, 256.84 (209.1) corresponding to the 2-month time-lag pattern in the testing phase for the January projection. SVM had the lowest RMSE and MAE for the February and March projections. SVM had the lowest RMSE (260.76), and RT had the lowest MAE (111.73) for the April projection. Appendix 6 confirms the accurate forecasting skill of SVM and RT for inflow to Gotvand reservoir with the 2-month time-lag pattern compared to the other forecasting algorithms.

RT has the lowest MAE for several projections with both time-lag patterns in the three reservoirs, while the minimal RMSE was obtained by SVM. It is seen in Appendixes 1–6 that RT calculated excellent forecasts for most years for the four projections; yet, RT had a large forecast error in some years. In contrast, SVM forecasted inflows with a relatively constant error. The MAE (Eq. (9)) calculates the mean of the absolute values of the differences between the observed and forecasted inflows to the reservoirs assigning the same weights to the differences. This is the main reason RT had lower MAE values than SVM under most projections, as RT forecasted most of the observed inflows well. On the other hand, the RMSE is the root of the mean square differences, which assigns more weight to the large differences because of the squaring applied [see Eq. (10)]. This caused SVM to produce lower RMSE than RT.

**Figure 7:**
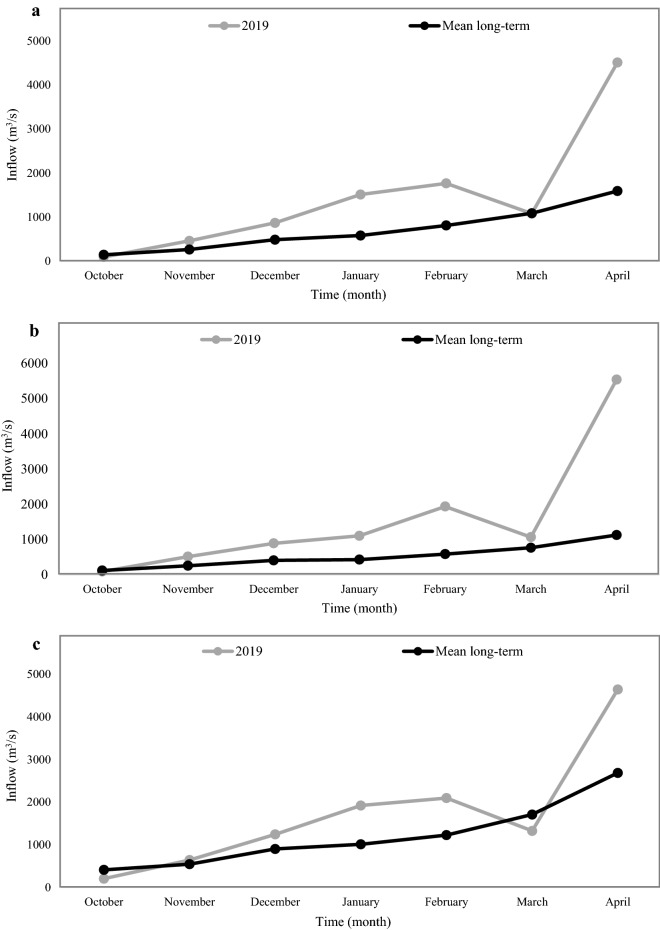
Mean long-term and 2019 reservoir monthly inflows: (**a**) Dez reservoir, (**b**) Karkheh reservoir and (**c**) Gotvand reservoir.

Tables 4, 5 and 6 establish that all the applied algorithms had the lowest forecasting accuracy under January projection with the 2-month time-lag pattern in the three reservoirs compared with the other projections judging by the significant drop in the values of the performance indices. This is so because the hydrologic or water year starts in September–October in Iran, and the algorithms for the January projection with a 2-month time lag forecast the reservoir inflows relying only on the October input data. It is evident in Fig. 7 that the reservoirs inflow in October 2019 are affected by the long-term reservoirs inflows and prolonged drought. Therefore, forecasting reservoirs inflow for the January projection with a 2-month time lag is more uncertain than the other projections.

A more detailed evaluation of the obtained results is the average improvement percentages (AIPs) of R and RMSE for the SVM and the AIPs of the MAE corresponding to the RT compared with the other forecasting algorithms in the testing phase when applying the 1-month and 2-month time-lag patterns. It is seen in Table 7 the clear superiority of the average R and RMSE associated with SVM model when using the 1-month time-lag pattern; that is, SVM features positive AIPs of R and RMSE when compared with RT, ANN, and GP. The largest AIPs of R and RMSE for SVM were obtained relative to ANN and GP (in Dez and Gotvand reservoirs), respectively. SVM featured negative AIPs of R compared to RT and GP in Dez reservoir and comparison with ANN, RT, and GP in the other reservoirs under the 2-month lag-time pattern, as shown in Table 7. Also, SVM had negative AIPs of RMSE in comparison with the RT in Karkheh and Gotvand reservoirs for the 2-month time-lag pattern. The reason for negative AIPs of R and RMSE for SVM was the SVM's performance decline with respect to the January projection with a 2-month time lag compared to the other algorithms in forecasting the reservoirs inflow. The most negative AIPs of R for SVM was obtained when compared with RT. Therefore, under the 2-month time-lag pattern, RT had higher accuracy on average than SVM, ANN and GP with respect to R. It is evident from Table 7 that RT had positive AIPs of MAE compared to SVM, ANN and GP except for the 2-month time-lag pattern in Gotvand reservoir. The largest positive AIPs of MAE for RT were obtained when compared with GP except for the 1-month time-lag pattern in the Karkheh reservoir.

**Table 7:** Average improvement percentages of R and RMSE with SVM, and of MAE with RT compared with the other algorithms in the testing phase.

Reservoir	Time-lag pattern	Algorithms	Top algorithms		
			SVM		RT
			R	RMSE	MAE
Dez	1-month	SVM	–	–	49.41
		ANN	47.50	72.00	85.32
		RT	3.56	13.62	–
		GP	13.00	72.71	86.00
	2-month	SVM	–	–	34.00
		ANN	9.75	47.40	71.57
		RT	− 10.00	16.67	–
		GP	− 1.00	49.33	77.65
Karkheh	1-month	SVM	–	–	9.25
		ANN	10.57	67.00	42.50
		RT	4.33	40.58	–
		GP	11.12	55.26	41.53
	2-month	SVM	–	–	53.00
		ANN	− 10.00	29.11	60.20
		RT	− 25.00	− 15.62	–
		GP	− 18.00	61.30	82.45
Gotvand	1-month	SVM	–	–	44.90
		ANN	22.25	74.00	75.28
		RT	8.00	29.54	–
		GP	10.80	71.28	84.34
	2-month	SVM	–	–	− 5.00
		ANN	− 5.60	32.00	61.77
		RT	− 20.10	− 0.50	–
		GP	− 9.20	68.00	85.35

### Evaluation of time-lag patterns

**Figure 8:**
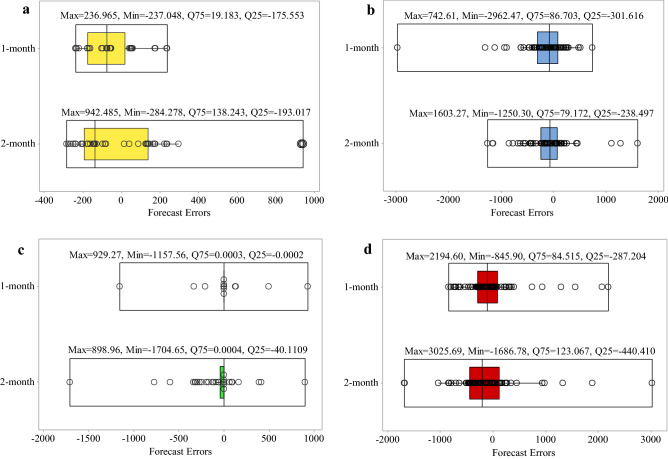
Boxplot of the algorithms’ error distribution in the testing phase for the 1-month and 2-month time-lag patterns in Dez resevoir: (**a**) SVM, (**b**) GP, (**c**) RT and (**d**) ANN.

**Figure 9:**
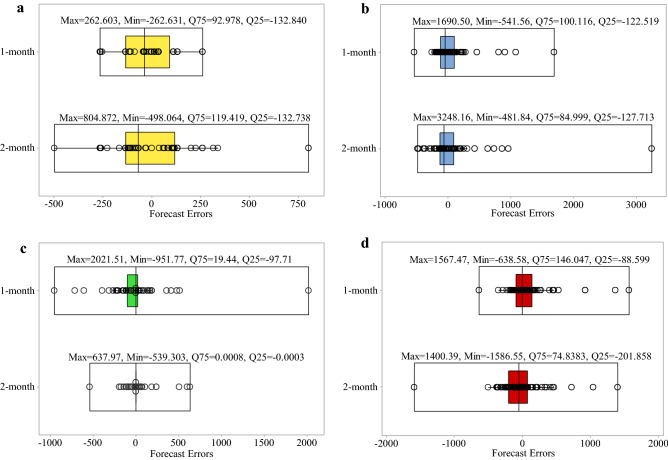
Boxplot of algorithms’ error distribution in the testing phase for the 1-month and 2-month time-lag patterns in Karkheh reservoir: (**a**) SVM, (**b**) GP, (**c**) RT and (**d**) ANN.

**Figure 10:**
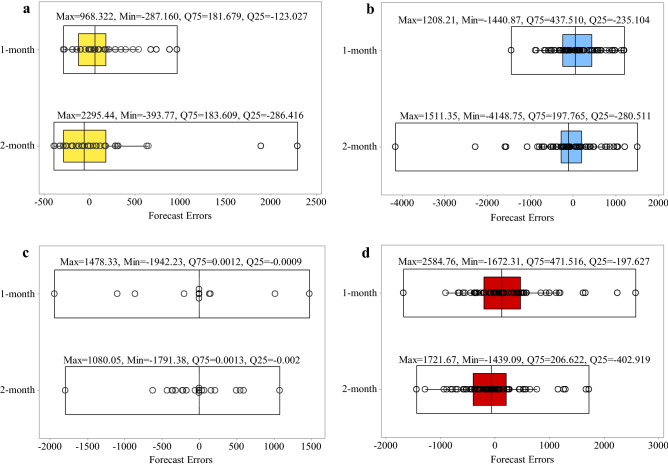
Boxplot of the algorithms’ error distribution in the testing phase for the 1-month and 2-month lag-time patterns in Gotvand reservoir: (**a**) SVM, (**b**) GP, (**c**) RT and (**d**) ANN.

The distribution of the forecast errors is examined with boxplots for further evaluation of the forecasting algorithms’ performance. The error equals the difference between the observed and forecasted inflows to the reservoirs. Positive and negative error values indicate under-estimation and over-estimation, respectively. The lower quartile (Q25) and upper quartile (Q75) contains one-fourth and three-fourths of the errors, respectively; therefore, the upper quartile is more significant than the lower quartile for comparing the algorithms’ performance. Figure 8a–d shows the SVM, GP, RT, and ANN results, respectively. It is seen that the upper quartiles for the 1-month time-lag pattern were equal to 19.183, 86.703, 0.0003, and 84.515, respectively, which were lower than the upper quartile for the 2-month time-lag pattern (138.243, 79.172, 0.0004, and 123.067, respectively), except GP. Therefore, SVM, RT, and ANN applying the 1-month time-lag pattern and GP applying the 2-month time-lag pattern had better accuracy in forecasting the inflow to the Dez reservoir. It is seen in Fig. 9 that the SVM’s upper quartile Q75 = 92.978 was more accurate for the 1-month time lag pattern; however, GP, RT, and ANN had Q75 = 84.991, 0.0008, and 74.838, respectively for the 2-month time-lag pattern performed better than the 1-month time-lag pattern in Karkheh reservoir. The minimum upper quartiles were equal to 181.679 and 0.0012 for SVM and RT, respectively, with the 1-month time-lag pattern, as can be seen in Fig. 10. GP and ANN, on the other hand, had better performance with the 2-month time-lag pattern based on the low values of their upper quartiles (equal to 197.765 and 206.622, respectively) in forecasting inflow to Gotvand reservoir.

### Evaluation of the performance of the applied algorithms in forecasting reservoirs inflow in 2019

**Figure 11:**
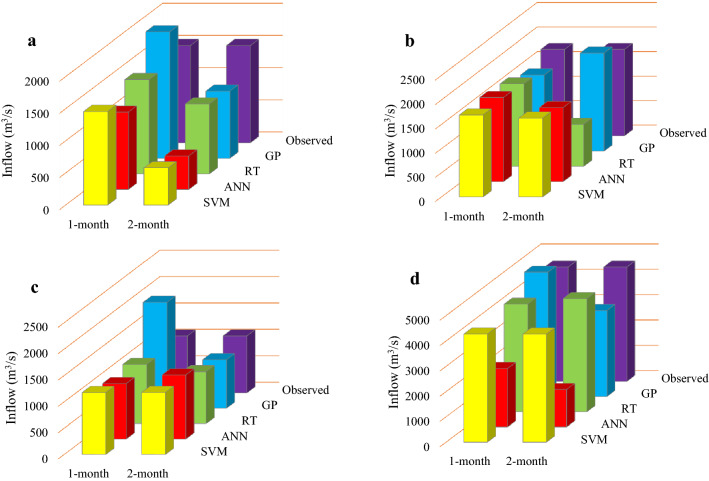
Forecasted inflow values of Dez reservoir in 2019 by the applied algorithms for 1- and 2-month time-lag patterns; (**a**) January projection, (**b**) Febuary projection, (**c**) March projection and (**d**) April projection.

**Figure 12:**
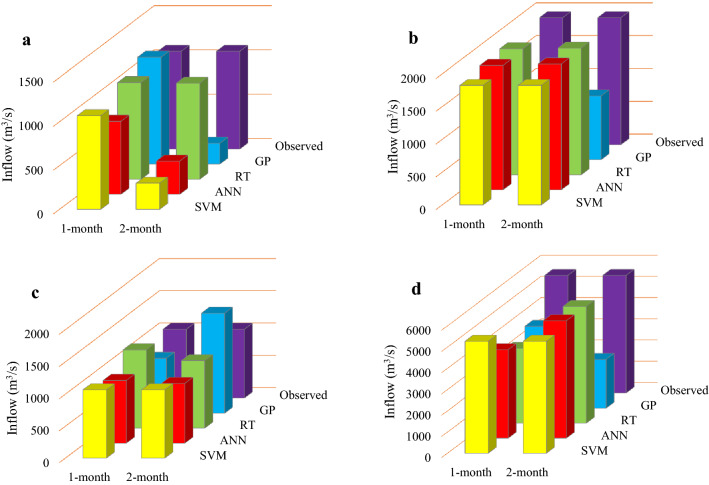
Forecasted inflow values of Karkheh reservoir in 2019 by the applied algorithms for 1- and 2-month time-lag patterns; (**a**) January projection, (**b**) Febuary projection, (**c**) March projection, and (**d**) April projection.

**Figure 13:**
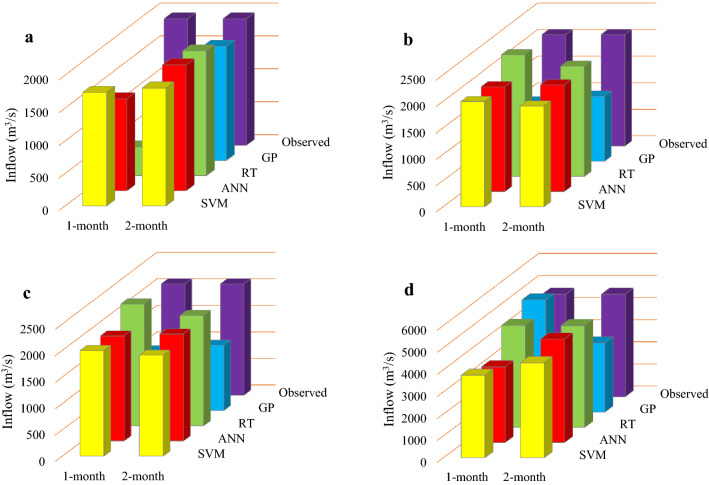
Forecasted inflow values of Gotvand reservoir in 2019 by the applied algorithms for 1- and 2-month time-lag patterns; (**a**) January projection, (**b**) Febuary projection, (**c**) March projection, and (**d**) April projection.

Figures 11, 12 and 13 display the performance of the applied forecasting algorithms corresponding to the 1- and 2-month time-lag patterns in foresting reservoirs inflow in 2019. As shown in Figs. 11, 12 and 13, the observed inflows to the Dez, Karkheh and Gotvand reservoirs in April and February 2019 are larger than the other months. A comparison of the observed reservoirs inflow reveals that the largest inflow in February accrues to Gotvand reservoir, and in April it corresponds to Karkheh reservoir.

#### Dez reservoir

It is seen in Fig. 11a that under the projection-January, RT and SVM with a 1-month time lag forecasted the Dez reservoir inflow with a lower error than the other algorithms and another time-lag pattern, which are 42.7 and 55.4 m3/s, respectively. Figure 11b,c show that ANN and RT with a 1-month time lag were more accurate in forecasting Dez reservoir inflows in February and March. The error values for the February projection are 32.7 and 71.1 m^3^/s, respectively, and for the March projection are 27.19 and − 44.31 m^3^/s, respectively. According to Fig. 11d the April projection with the RT model obtained with a 2-month time lag has an error of 38.0 m^3^/s, and SVM model with a 1-month time lag has an error of 236.6 m^3^/s.

#### Karkheh reservoir

Comparison of the forecasted inflows to Karkheh reservoir in 2019 shows that the RT model with 1- and 2-month time lag for the January projection (with errors equal to 6.9 and 13.9 m^3^/s, respectively) and the February projection (with errors equal to 1.2 and 5.0 m^3^/s, respectively) is superior to the other algorithms (see Fig. 12a,b). The minimum forecasts error of Karkheh reservoir inflows for the March projection belongs to RT with a 2-month time lag, and to ANN with a 1-month time lag (with errors equal to 89.4 and 149.7 m^3^/s, respectively) and for the April projections belongs to RT and ANN with a 2-month time lag (with errors equal to 3.2 and 6.7 m^3^/s, respectively).

#### Gotvand reservoir

Figures 13a,b show the superiority of RT and ANN for the 2-month time-lag pattern for the January projection (with errors equal to 22.4 and 1.2 m^3^/s, respectively) and for the February projection (with errors equal to 15.8 and 74.2 m^3^/s, respectively) in forecasting Gotvand reservoir inflows in 2019 compared to the other applied algorithms and another time-lag pattern. Furthermore, RT with 1- and 2- month time lag for the March projection (with errors equal to 39.1 and 31.8 m^3^/s, respectively) and for the April projection (with errors equal to 18.4 and 29.1 m^3^/s, respectively) had better performance accuracy in forecasting Gotvand reservoir inflows according to Fig. 13c,d.

## Concluding remarks

This study presents a method for forecasting reservoirs inflow. SVM, ANN, RT, and GP were selected to forecast the monthly inflows to Dez, Karkheh, and Gotvand reservoirs in Iran. The proposed method is applied to evaluate the forecasting performance of the algorithms during the large flood of 2019. The applied algorithms were developed based on the 1-month and 2-month time-lag patterns. Monthly reservoirs inflow were used to train the forecasting algorithms. The forecasting skill of the algorithms were compared using the Correlation Coefficient, Root Mean Squared Error, Nash–Sutcliffe efficiency, and Mean Absolute Error. The capacity of RT to forecast the largest reservoir inflows in 2019 indicates that the reservoir inflows in 2019 could have been forecasted accurately. The results showed that SVM and RT had better accuracy among the algorithms. The SVM model with the 1-month time-lag pattern performed better (22.14%) than the 2-month time-lag pattern according to the upper quartile (Q75) of forecast errors distribution in forecasting the Karkheh reservoir’s inflow. In contrast, the RT model had better accuracy (99%) with the 2-month time-lag pattern. Furthermore, SVM and RT had better performance with the 1-month time lag based on the low value of Q75 in forecasting inflow to Dez (86.12 and 25%, respectively) and Gotvand (1 and 7.69%, respectively) reservoirs.

This study’s results guide FIRO for improved reservoir management, decision-making and planning, and optimal reservoir storage allocation for flood control. Accurate forecasting of reservoir inflow is imperative for effective and timely flood control, reduction of damages, and for reducing the risk of not meeting downstream water demands.

Future research may be applied to develop ensemble models and comparing their performance with the ML algorithms in forecasting the 2019 reservoir inflows. Furthermore, comparing the forecasting skill of the ML algorithms with those of physically-based models for forecasting reservoir inflows would provide a comprehensive assessment of the relative advantages of these forecasting methods. Employing remote sensing data in data-sparse areas, especially for developing countries, would be worth pursuing in future works.

## Supplementary Information

Supplementary Information.

## Data Availability

The data that support the findings of this study are available from the corresponding author upon reasonable request.

## Code availability

The codes that support the findings of this study are available from the corresponding author upon reasonable request.
