# Supplementary material for: Machine-learning algorithms for forecast-informed reservoir operation (FIRO) to reduce flood damages
Source: Sci Rep. 2021 Dec 21;11:24295. doi: 10.1038/s41598-021-03699-6 (PMC8692612; doi:10.1038/s41598-021-03699-6)
Supplement: Supplementary file 1 — Supplementary Information. [file 41598_2021_3699_MOESM1_ESM.docx]

**Appendixes**

**
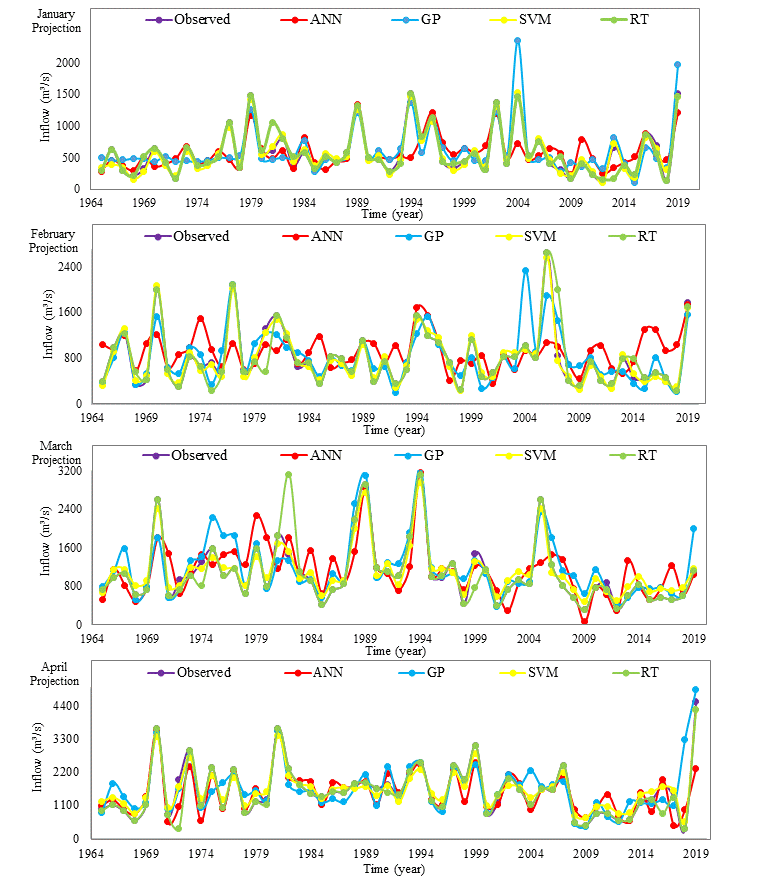
Appendix 1.** Results of the applied algorithms obtained with the 1-month time-lag pattern in Dez reservoir for the four projections (ANN, GP, SVM, RT)**.**

**
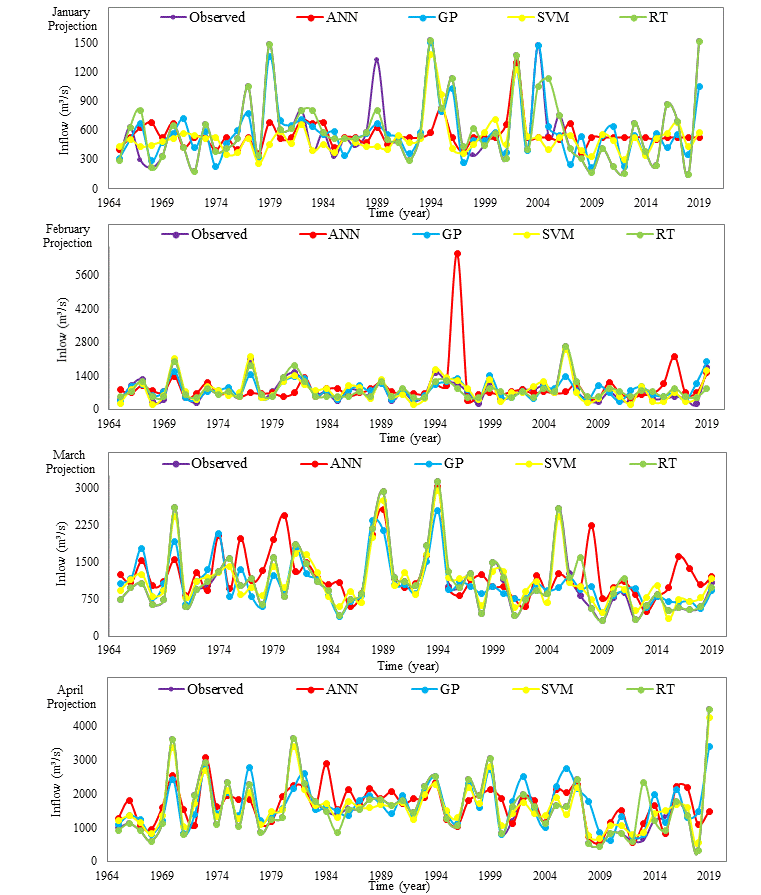
Appendix 2.** Results of the applied algorithms obtained with the 2-month time-lag pattern in Dez reservoir for the four projections (ANN, GP, SVM, RT)**.**

**
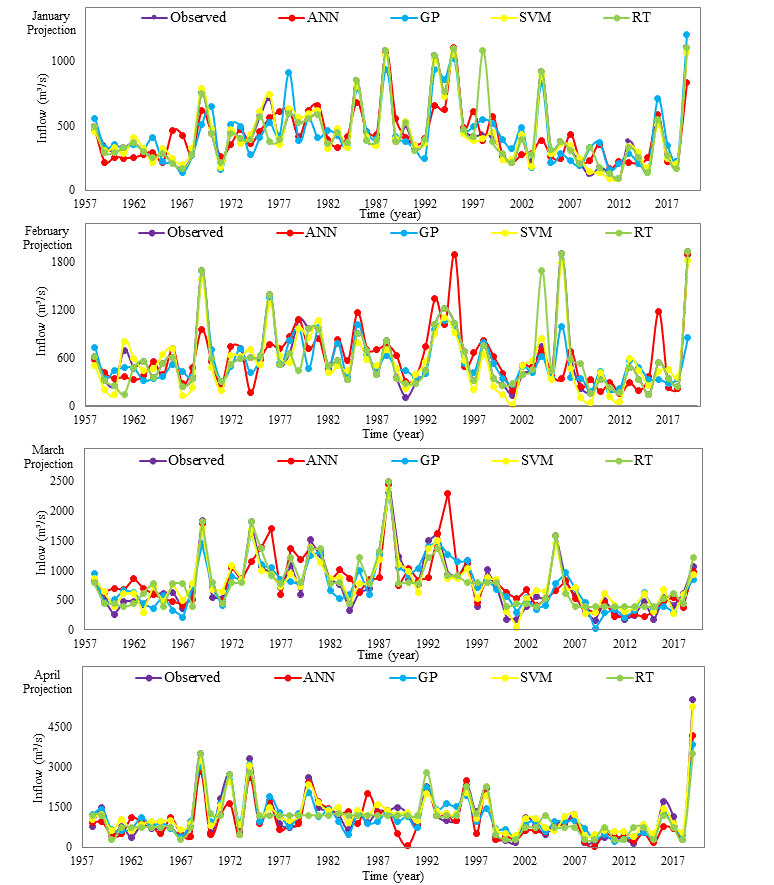
Appendix 3.** Results of the applied algorithms obtained with the 1-month time-lag pattern in Karkheh reservoir for the four projections (ANN, GP, SVM, RT)**.**

**
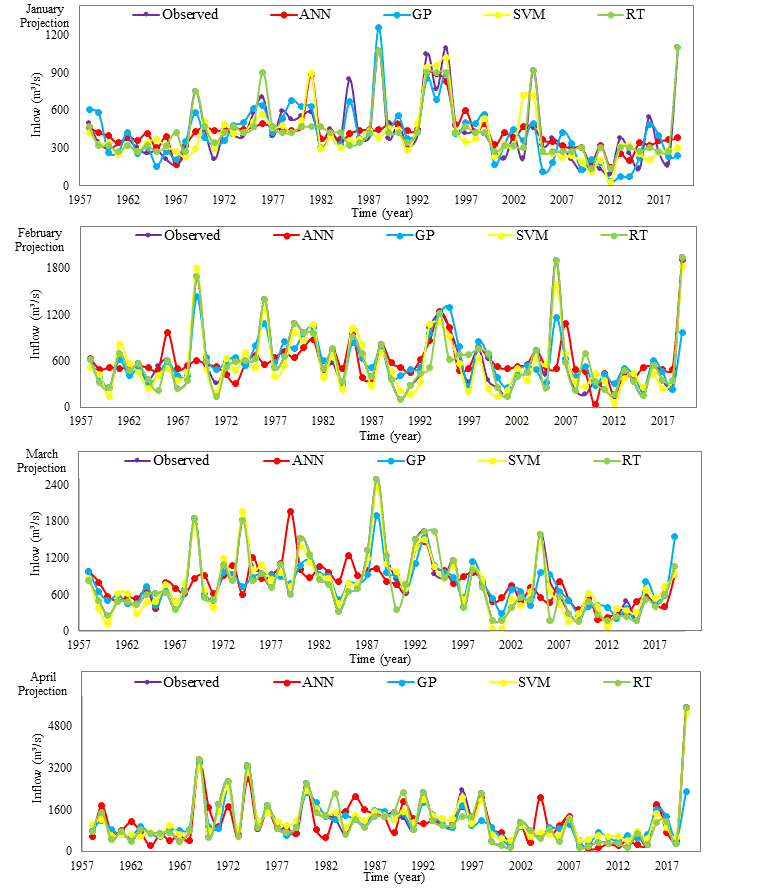
Appendix 4.** Results of the applied algorithms obtained with the 2-month time-lag pattern in Karkheh

reservoir for the four projections (ANN, GP, SVM, RT)**.**


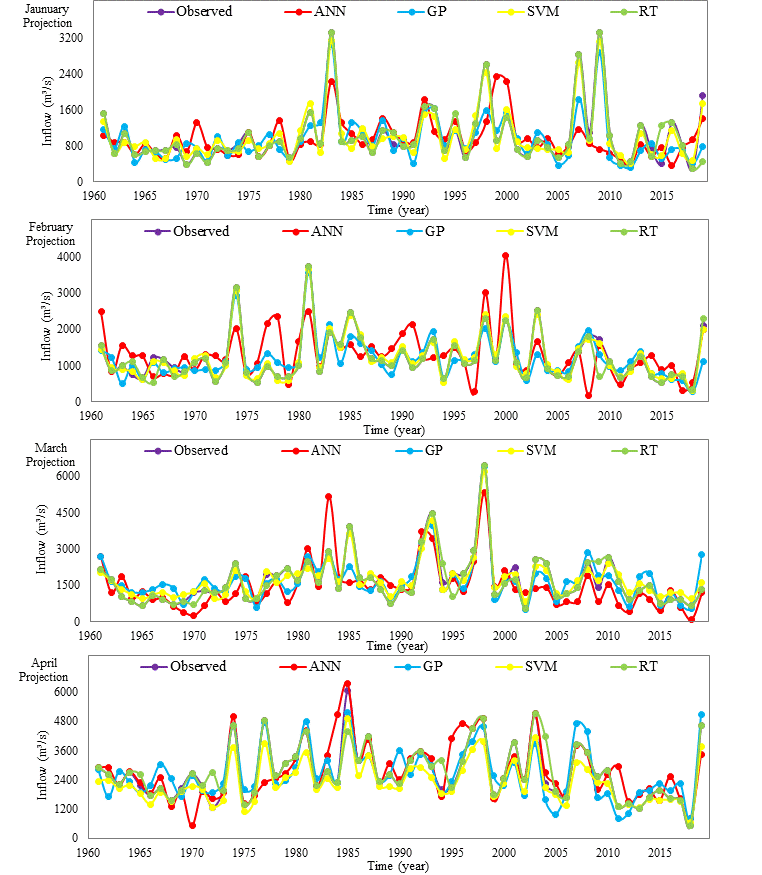
**Appendix 5.** Results of the applied algorithms obtained with the 1-month time-lag pattern in Gotvand reservoir for the four projections (ANN, GP, SVM, RT)**.**

**
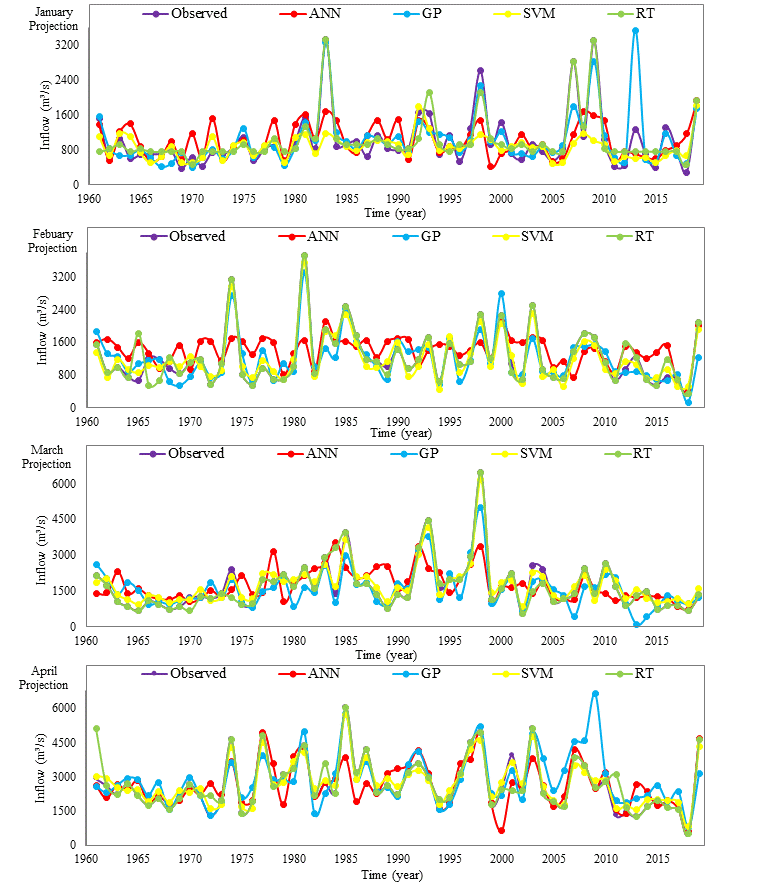
Appendix 6.** Results of the applied algorithms obtained with the 2-month time-lag pattern in Gotvand reservoir for the four projections (ANN, GP, SVM, RT)**.**
